# Macadamia germplasm and genomic database (MacadamiaGGD): A comprehensive platform for germplasm innovation and functional genomics in *Macadamia*

**DOI:** 10.3389/fpls.2022.1007266

**Published:** 2022-10-27

**Authors:** Pan Wang, Yi Mo, Yi Wang, Yuchong Fei, Jianting Huang, Jun Ni, Zeng-Fu Xu

**Affiliations:** ^1^ State Key Laboratory for Conservation and Utilization of Subtropical Agro-Bioresources, College of Forestry, Guangxi University, Nanning, China; ^2^ Key Laboratory of National Forestry and Grassland Administration for Fast-Growing Tree Breeding and Cultivation in Central and Southern China, College of Forestry, Guangxi University, Nanning, China

**Keywords:** *Macadamia*, germplasm, genome, SSR, SNP, MacadamiaGGD, genomic database, molecular breeding

## Abstract

As an important nut crop species, macadamia continues to gain increased amounts of attention worldwide. Nevertheless, with the vast increase in macadamia omic data, it is becoming difficult for researchers to effectively process and utilize the information. In this work, we developed the first integrated germplasm and genomic database for macadamia (MacadamiaGGD), which includes five genomes of four species; three chloroplast and mitochondrial genomes; genome annotations; transcriptomic data for three macadamia varieties, germplasm data for four species and 262 main varieties; nine genetic linkage maps; and 35 single-nucleotide polymorphisms (SNPs). The database serves as a valuable collection of simple sequence repeat (SSR) markers, including both markers that are based on macadamia genomic sequences and developed in this study and markers developed previously. MacadamiaGGD is also integrated with multiple bioinformatic tools, such as search, JBrowse, BLAST, primer designer, sequence fetch, enrichment analysis, multiple sequence alignment, genome alignment, and gene homology annotation, which allows users to conveniently analyze their data of interest. MacadamiaGGD is freely available online (http://MacadamiaGGD.net). We believe that the database and additional information of the SSR markers can help scientists better understand the genomic sequence information of macadamia and further facilitate molecular breeding efforts of this species.

## Introduction

Macadamia (*Macadamia* spp.), which belongs to the Proteaceae family (Urata, 1954), is an evergreen perennial flowering plant species (Storey and Hamilton, 1953) originating from southern Queensland and northern New South Wales in Australia (Moncur et al., 1985). Macadamia has already become one of the most important economic oil crop species worldwide (Sedgley, 1983; Aradhya et al., 1998; Topp et al., 2019) due to the high level of monounsaturated fatty acid-palmitoleic acid (omega-7) in its nuts, which can effectively lower blood total cholesterol and benefit human health (Nagao et al., 1992; Moodley et al., 2007; Arroyo-Caro et al., 2016). To date, four macadamia species, namely, *Macadamia integrifolia* (Maiden & Betche), *M. tetraphylla* (L. A. S. Johnson), *M. ternifolia* (F. Muell), and *M. jansenii* (C.L. Gross & P.H. Weston), have been identified (Mast et al., 2008), among which only *M. integrifolia*, *M. tetraphylla*, and their hybrids are most widely planted worldwide (SAMAC, 2020). The other two species, *M. ternifolia* and *M. jansenii*, have not yet been used for any commercial purpose because they produce only small, unpalatable, bitter, inedible nuts, the mature nuts of which contain high cyanogenic glycoside levels (Trueman, 2013; Mai et al., 2020).

Macadamia plants are diploid (2n = 28) (Peace et al., 2003) and their genome size ranges from 758 to 896 megabase (Mb) (Nock et al., 2020; Niu et al., 2022a). In recent years, several *de novo*-assembled macadamia genomes have been reported, providing new insight for genetic breeding. In 2016, the first assembled draft genome of macadamia (*M. integrifolia* cultivar HAES 741) was finished and released by Nock’s lab, the staff of whom used the short-read Illumina sequence platform (193493 scaffolds, N50 = 4745 bp, 518 Mb) (Nock et al., 2016). In 2020, the first sequence-based genetic linkage maps of macadamia were constructed (Langdon et al., 2020). In 2020, an improved chromosome-scale genome assembly of *M. integrifolia* cultivar HAES 741 was completed by the use of the short-read Illumina and long-read Pacific Biosciences (PacBio) sequencing platforms (4094 scaffolds, N50 = 413 kb, 745 Mb) (Nock et al., 2020). Furthermore, in 2020, by using the third-generation sequencing (TGS) platforms Oxford Nanopore (PromethION), PacBio (Sequel I), and BGI (Single-tube Long Fragment Read), researchers assembled the genome of *M. jansenii* (Murigneux et al., 2020). In addition, the genomes of *M. integrifolia* (249 contigs, N50 = 5.3 Mb, 738 Mb), *M. tetraphylla* (153 contigs, N50 = 10.0 Mb, 707 Mb), *M. ternifolia* (211 contigs, N50 = 6.4 Mb, 716 Mb), and *M. jansenii* (284 contigs, N50 = 4.5 Mb, 738 Mb) were assembled by use of the PacBio HiFi TGS platform (Sharma et al., 2021a). The genome of *M. jansenii* has been improved by Hi-C assembly (219 scaffolds, N50 = 52 Mb, 758 Mb) (Sharma et al., 2021c) and was further updated by the latest hifiasm assembly (779 contigs, N50 = 46 Mb, 826 Mb) (Sharma et al., 2021b). Recently, the genome of the cultivar HAES 344 was sequenced and assembled into 14 pseudochromosomes by the use of Illumina NovaSeq and PacBio Sequel II sequencing (5387 contigs, N50 = 281 kb, 794 Mb) (Lin et al., 2022). A chromosome-scale genome assembly of *M. tetraphylla* has also been constructed from long-read Oxford Nanopore Technologies (ONT) sequencing data (1059 scaffolds, N50 = 51 Mb, 751 Mb) (Niu et al., 2022a). Moreover, in recent years, the chloroplast and mitochondrion genomes of *M. integrifolia*, *M. tetraphylla*, and *M. ternifolia* have been assembled and thoroughly annotated (Niu et al., 2022b).

As inbreeding decline occurs in macadamia, it is vitally important to understand the genetic distances between individuals (Steiger et al., 2003). The morphological characteristics of macadamia could be greatly influenced by the environment; thus, it is sometimes difficult to identify genetic relationships through phenotypic observations (Hardner, 2016). The use of DNA marker systems has become one of the most efficient strategies to evaluate genetic distance and genetic foundation (Ranketse et al., 2022). DNA marker systems, including isozyme (Vithanage and Winks, 1992; Aradhya et al., 1998), randomly amplified DNA fingerprinting (RAF) (Peace et al., 2002; Peace et al., 2004; Peace et al., 2005), amplified fragment length polymorphism (AFLP) (Steiger et al., 2003), sequence tagged site (STS) (Vithanage et al., 1998), random amplified polymorphic DNA (RAPD) (Vithanage et al., 1998), randomly amplified microsatellite fingerprinting (RAMiFi) (Peace et al., 2004), simple sequence repeat (SSR) (Schmidt et al., 2006; Nock et al., 2014b; Langdon et al., 2019; Ranketse et al., 2022), diversity array technology (DArT) and single-nucleotide polymorphism (SNP) markers (Alam et al., 2018; O'Connor et al., 2019b), have been developed for the genetic and molecular breeding of macadamia. Genome-wide association studies (GWASs) have also greatly facilitated the identification of new molecular markers associated with yield traits (O'Connor et al., 2019a; O'Connor et al., 2020). As codominant, highly reproducible, highly polymorphic and cost-efficient DNA markers, SSRs have been preferred for use in studies of genetic identification and diversity analysis. To date, although the sequencing of the whole genomes of different macadamia species has been completed, genome-based development of SSR markers has not been reported.

With the rapidly developed sequencing technologies, the genomes of dozens of plant species have been sequenced each year. Nevertheless, how to integrate and well manage the large amount of omics data is still a task. In recent years, the genomic databases of some economic crops were well constructed and greatly facilitated the researchers to use the genome, transcriptome, or phenotype data. Citrus Genome Database (CGD, https://www.citrusgenomedb.org/) integrates genomes, maps, markers, phenotype data, and quantitative trait loci of agronomic traits of 25 citrus species. The Rice Genome Hub (RGH, https://rice-genome-hub.southgreen.fr), which is part of the South Green Bioinformatics platform, also integrates large amount of rice omics data with a large number of powerful in-house tools (Droc et al., 2019). Rice Annotation Project Database (RAP-DB, https://rapdb.dna.affrc.go.jp/) is consisted of updated genome annotation and focuses on the comprehensive analysis of genome structure and function of rice genes (Project, 2007). Gossypium Resource and Network Database (GRAND, http://grand.cricaas.com.cn) contains the genomic, transcriptomic, phenotypic, and integrative analysis tools for cotton (Zhang et al., 2022). With the inspirations from these databases, in this study we developed the first integrated germplasm and functional genomic database for macadamia (MacadamiaGGD).

Currently, large amounts of macadamia omics data lack centralized management. These data are distributed across multiple repositories or personal websites, with the same data from the same source in different repositories. In addition, many macadamia omics data lack the management of versions. The same data has different versions and accession numbers in different repositories, which can make it difficult for users to find the most updated dataset. The main purpose of the MacadamiaGGD described in this article is to provide the germplasm data, genome resources, transcriptome (RNA-seq) data, molecular marker information and genetic linkage map information to assist in the scientific research and molecular breeding of macadamia. And several commonly used bioinformatics tools are also integrated with MacadamiaGGD, which can help the researchers better utilize the database.

## Materials and methods

### Data sources and processing

**Figure 1:**
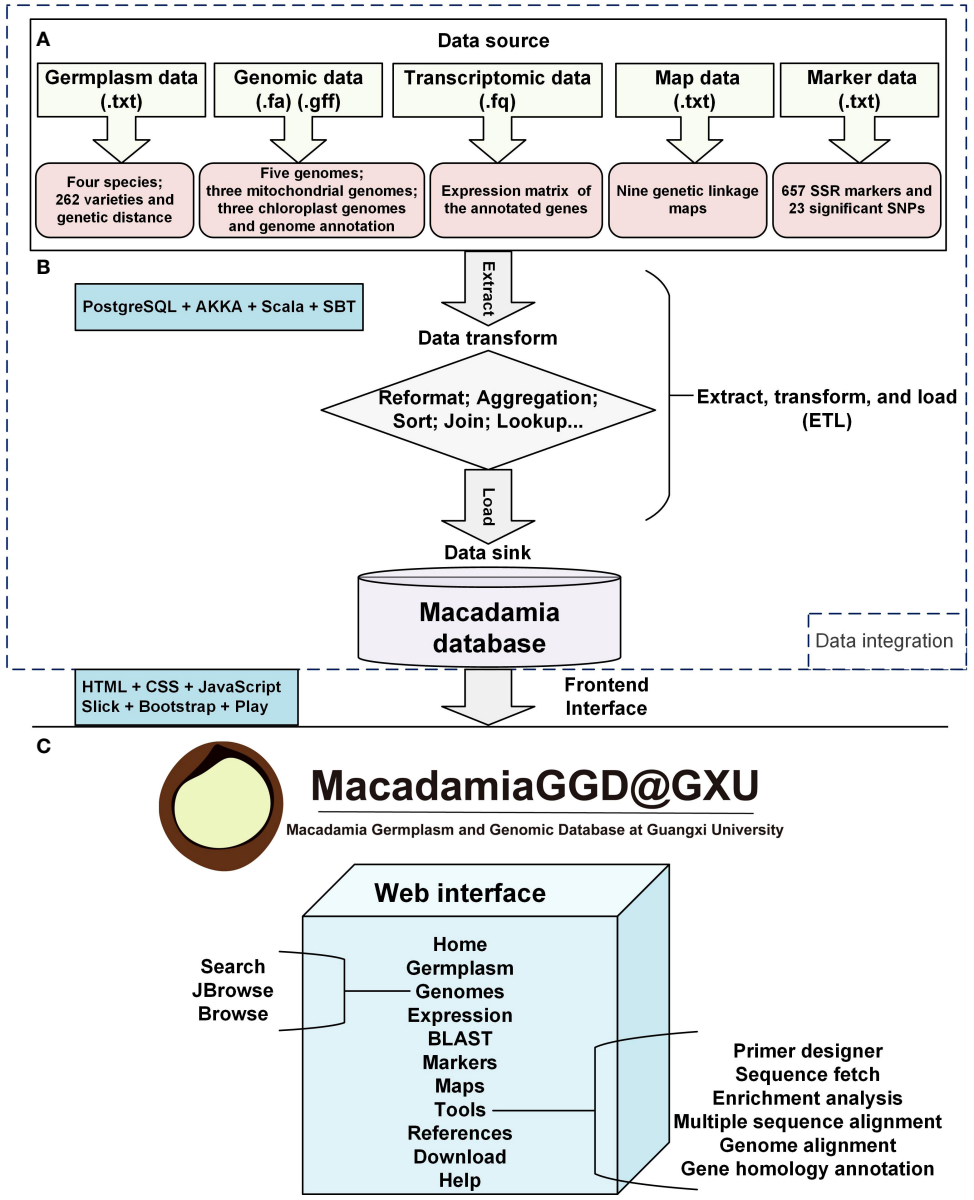
Feature diagram of MacadamiaGGD. MacadamiaGGD is a collection of germplasm, genomic, transcriptomic, maps, and molecular marker data of macadamia, and multiple bioinformatic tools. All the data are stored and managed in a PostgreSQL database. **(A)**, Data source layer. **(B)**, Middleware layer. **(C)**, Application layer.

In MacadamiaGGD, we integrated the genetic information data, including that of five genomes of four species, the chloroplast and mitochondrion genomes of three species and genome annotations, which were previously released in public databases, including the National Center for Biotechnology Information (NCBI) Assembly database, the *GigaScience* database (*Giga*DB), and the China National Center for Bioinformation (CNCB) Genome Warehouse (GWH) database. In addition, transcriptomic data for three macadamia varieties were downloaded from the NCBI Sequence Read Archive (SRA) database. The germplasm, genetic linkage map, SNP and SSR marker data were retrieved from the NCBI PubMed database and other databases, as summarized in **Table 1**. The components of data integration mainly include the data source, the data transform, and the data sink in the database. Extract, transform, and load (ETL) architecture was applied to data integration. In data integration process, raw data were collected, transformed, sorted, cleaned, aggregated, and stored *via* using PostgreSQL 9.5.25, Scala 2.13.1, AKKA 2.6.5, and SBT 1.3.5 (**Figures 1A, B**). Processed raw data were applied for variation calling and data visualization though using HTML5, CSS3, Java Script, Slick 3.3.2, Bootstrap 3.3.0 and Play Framework 2.8.2 (**Figure 1B**).

**Table 1:** Summary of all datasets in MacadamiaGGD.

Dataset	Species	References	Repository/Accession number	URL
Germplasm	*M. integriflia* *M. ternifolia* *M. tetraphylla* *M. jansenii*	Vithanage and Winks (1992); Aradhya et al. (1998); Peace et al. (2002); Peace et al. (2005); Allan (2007); He (2008); Gitonga et al. (2009); Hardner et al. (2009); Machado Neto and Moryia (2010); Zhang (2011); Hardner (2016); Zeng and Du (2017); Alam et al. (2018); Tang et al. (2018); Toft et al. (2018); Langdon et al. (2019); O'Connor et al. (2019b); Tan et al. (2019); Tan et al. (2020); Mai et al. (2021); Tan et al. (2021); Lin et al. (2022)		http://MacadamiaGGD.net/nut/toRef
Genome assembly	*M. integriflia* HAES 741	Nock et al. (2020)	NCBI/PRJNA748012	https://www.ncbi.nlm.nih.gov/bioproject/748012
	*M. integriflia* HAES 344	Lin et al. (2022)	CNCB/PRJCA004595	https://ngdc.cncb.ac.cn/gwh/Assembly/23196/show
	*M. tetraphylla*	Sharma et al. (2021a)	GigaDB/100906; NCBI/PRJNA694456	http://gigadb.org/dataset/view/id/100906/
	*M. ternifolia*	Sharma et al. (2021a)	GigaDB/100906; NCBI/PRJNA694456	http://gigadb.org/dataset/view/id/100906/
	*M. jansenii*	Sharma et al. (2021a)	GigaDB/100906; NCBI/PRJNA694456	http://gigadb.org/dataset/view/id/100906/
Genome annotation	*M. integriflia* HAES 741	Nock et al. (2020)	NCBI/PRJNA748012	https://www.ncbi.nlm.nih.gov/bioproject/748012
	*M. integriflia* HAES 344	Lin et al. (2022)	CNCB/PRJCA004595	https://ngdc.cncb.ac.cn/gwh/Assembly/23196/show
Chloroplast assembly and annotation	*M. integriflia*	Nock et al. (2014a)	NCBI/PRJNA264682	https://www.ncbi.nlm.nih.gov/genome/?term=txid60698
	*M. ternifolia*	Liu et al. (2017)	NCBI/PRJNA421511	https://www.ncbi.nlm.nih.gov/genome/browse/#!/organelles/66349/
	*M. tetraphylla*	Liu et al. (2018)	NCBI/MH778649	https://www.ncbi.nlm.nih.gov/nuccore/MH778649
Mitochondrion assembly and annotation	*M. integriflia*	Niu et al. (2022b)	NCBI/MW566570	https://www.ncbi.nlm.nih.gov/nuccore/MW566570
	*M. ternifolia*	Niu et al. (2022b)	NCBI/MW566571	https://www.ncbi.nlm.nih.gov/nuccore/MW566571
	*M. tetraphylla*	Niu et al. (2022b)	NCBI/MW566572	https://www.ncbi.nlm.nih.gov/nuccore/MW566572
Transcriptome	*M. integriflia* HAES 741	Nock et al. (2020)	NCBI/PRJNA593881	https://www.ncbi.nlm.nih.gov/bioproject/PRJNA593881
	*M. integriflia* HAES 344	Lin et al. (2022)	NCBI/PRJNA706119	https://www.ncbi.nlm.nih.gov/bioproject/PRJNA706119
	*M. integriflia* H2	Lin et al. (2022)	NCBI/PRJNA706119	https://www.ncbi.nlm.nih.gov/bioproject/PRJNA706119
Genetic linkage maps	*M. integriflia*	Langdon et al. (2020)		https://researchportal.scu.edu.au/esploro/outputs/dataset/991012821025202368
SSRs	*M. integriflia*	Schmidt et al. (2006); Nock et al. (2014b); Langdon et al. (2019)		http://MacadamiaGGD.net/nut/toRef
SNPs	*M. integriflia*	O'Connor et al. (2019a); O'Connor et al. (2020)		http://MacadamiaGGD.net/nut/toRef
	*M. integriflia* *M. ternifolia* *M. integriflia* **×** *M. tetraphylla* *M. jansenii*	(Alam et al., 2018)		

The datasets were deposited in the repositories of the China National Center for Bioinformation (CNCB), the National Center for Biotechnology Information (NCBI), and the GigaDB.

### Development of the database

MacadamiaGGD was deployed in the Ubuntu 16.04 operation system using AKKA 2.6.5 (https://akka.io) as the web server, PostgreSQL 9.5.25 (https://www.postgresql.org) as the database server, Scala 2.13.1 (https://www.scala-lang.org) as the programming language and SBT 1.3.5 (https://www.scala-sbt.org) as the interactive building tool. All the data were managed and stored in the PostgreSQL Database. The website interface was generated *via* Bootstrap 3.3.0 (https://getbootstrap.com) and Play Framework 2.8.2 (https://www.playframework.com/). The web interface of MacadamiaGGD was developed using HTML5, CSS3, Java Script. The query function was enforced based on the Slick 3.3.2 middleware tier. JBrowse 1.16.6 (https://www.jbrowse.org) was used for genome visualization.

### Sample collection and DNA isolation

Leaf samples of 21 macadamia accessions for DNA isolation were collected from the macadamia plantation in Chongzuo, Guangxi, China (**Table S1**). The DNA was isolated following a previously described method (Doyle, 1991), with slight modifications. To avoid problems of low efficiency and insufficient grinding due to manual grinding, young leaves were ground in a Tissuelyser-192 (Shanghai Jingxin Industrial Development Co., Ltd., China) and extracted with a 2% cetyltrimethylammonium bromide (CTAB) buffer. Nucleic acids were isolated with a chloroform: isoamyl alcohol (24:1) solution. DNA was purified with ethanol and resuspended in sterile distilled water. The DNA quality and concentration were assessed using ultraviolet spectrometry *via* a Nanodrop 2000c (Thermo Fisher Scientific, MA, USA) and agarose gel electrophoresis. The purified DNA was stored at -20°C until use.

### Genome-wide SSR screening and characterization

New microsatellite markers were screened in the *M. integrifolia* HAES 741 reference genome (https://www.ncbi.nlm.nih.gov/bioproject/748012) by using SSRHunter 1.3 (http://www.bio2soft.net) (Li and Wan, 2005). The search criteria were set as 2, 3, and 4 nucleotides, corresponding to at least 4 repetitions. Afterward, the SSRs, comprising no fewer than 30 repeated motifs and being evenly distributed on each chromosome, were preferentially selected. To further confirm the quality of the SSR markers, each sequence was again queried *via* BLAST within MacadamiaGGD and tested *via* polymerase chain reaction (PCR).

Primer 3 (https://primer3.org) was used to design primer pairs flanking the sequences of the screened SSR motifs. The primer design parameters were as follows: primer length, 17-25 bp; melting temperature (Tm), 53 °C; amplicon size, 350-500 bp; and GC content, 40-60%.

### Marker analysis, data analysis and map construction

The SSR PCR mixture (10 μL) comprised 1 μL of DNA, 0.4 μL of each primer (10 μM), 5 μL of Rapid Taq Master Mix (Vazyme, China) and 3.2 μL of double-distilled water. The amplification reaction program was as follows: 5 min at 95°C; 36 cycles of (30 s at 95°C, 53°C and 72°C); and a final extension of 5 min at 72 °C. Afterward, the mixture was held at 16°C. The PCR products were examined by electrophoresis on a 7% nondenaturing polyacrylamide gel run at 220 V for 40 min and visualized by silver staining. The density distribution map of polymorphic SSR markers on chromosomes was generated using MG2C software (http://mg2c.iask.in/mg2c_v2.1/).

## Results

### Overview of MacadamiaGGD

MacadamiaGGD contains the most comprehensive bioinformatics datasets of macadamia (including five genomes, a total of 89.28 Gb of transcriptomic data, three chloroplast and mitochondrion genomes, germplasm data for four species and 262 main varieties, nine genetic linkage maps, 35 SNPs and 657 SSR markers), which provides convenient access to the large amount of germplasm and genomic information of macadamia (**Figure 1A**). MacadamiaGGD is composed of 11 main functional modules: Home, Germplasm, Genomes, Expression, BLAST, Markers, Maps, Tools, References, Download and Help (**Figure 1C**). MacadamiaGGD can be used to search and visualize genomic information by using various tools, including search, JBrowse, BLAST, primer designer, sequence fetch, enrichment analysis, multiple sequence alignment, genome alignment, and gene homology annotation (**Figure 1**). MacadamiaGGD also provides information about macadamia germplasm and genome-related references. In summary, researchers can use the above functional modules of the database to quickly acquire the germplasm and genomic information of macadamia.

### Germplasm

In the Germplasm module of MacadamiaGGD, 23 agronomic traits of four species and 16 agronomic traits of 262 main varieties were carefully described, including tree vigor, leaf type, fruit shape, flower color, the early-bloom stage and full-boom stage, and others. Users can easily obtain information on the morphological characteristics of four macadamia species and 262 varieties in the germplasm module. In addition, a phylogenetic analysis tool based on the results of Alam et al. (2018), which shows genetic distances between individuals genotypes, is provided in this module.

### Genome browse and search

**Figure 2:**
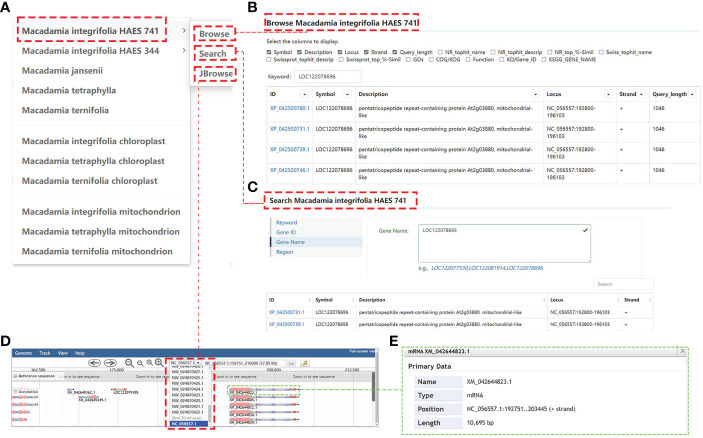
General view of the “Genomes” module. **(A)**, The genome module includes “11 macadamia genomes”, and three tools including “Browse”, “Search”, and “JBrowse”. **(B)**, The Browse information of gene “*LOC122078696*” in *Macadamia integrifolia* HAES 741 genome. **(C)**, Showing the Search result of gene “*LOC122078696*”. **(D)**, The JBrowse information of gene “*LOC122078696*”. **(E)**, Detailed description interface of mRNA XM_042644823.1.

The MacadamiaGGD database provides public information on the assembled genomes of the *M. integrifolia*, *M. tetraphylla*, *M. ternifolia*, and *M. jansenii*, which are available in different public databases. For example, when “Genomes” is clicked on, the column header label appears, showing the suboptions as in **Figure 2A**. We can choose any label to access the sublinks and search for the needed information. When the user enters a gene “*LOC122078696*” in *Macadamia integrifolia* HAES 741 genome Browse, it will get the structure and function annotation information of all transcript of the gene (**Figure 2B**). Moreover, when the user clicks “Search”, a new layer appears with four options: “Keyword”, “Gene ID”, “Gene Name”, and “Region” (**Figure 2C**). Then, if one clicks “Gene ID”, the interface appears as a blank box (**Figure 2C**). The user can enter the gene “*LOC122078696*” in the box and click the Search button; then, the requested information is displayed (**Figure 2C**).

### Genome JBrowse

Gene annotations in MacadamiaGGD are displayed graphically in the genome JBrowse, which includes the information of the gene location, nucleotide sequences, amino acid sequences, and other features. For example, if a user selects the genomic region from 192751 bp to 203445 bp on Chromosome 14 (NC_056557.1) for browsing, all genes located within this zone are displayed properly (**Figure 2D**). Further, when the mRNA XM_042644823.1 is clicked on, detailed information on its mRNA, coding sequence (CDS), and other features are displayed (**Figure 2E**).

### Transcriptomes of macadamia from different tissues

In the expression module of MacadamiaGGD, a total of 89.28 Gb of raw RNA-seq data were collected from tissues of young leaves, shoots, and flowers from the cultivar ‘Mauka’ (Nock et al., 2020); tissues of leaves, stems, flowers, and roots from the cultivar ‘Kau’; and shells and kernels at five different development stages from cultivar ‘Hinde’ (Lin et al., 2022). By mapping the transcriptome data to the reference genome and using transcripts per million (TPM) for calculation, we acquired the expression matrix of the annotated genes of macadamia.

### BLAST

**Figure 3:**
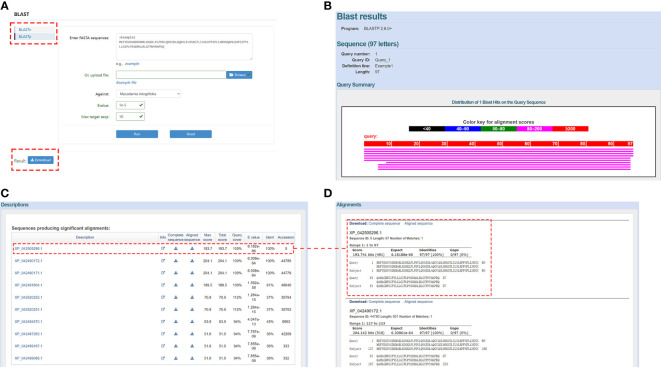
View of the “BLAST” module. **(A)**, Demonstration of the “BLASTp” box. **(B)**, Example of the search result after a sequence was input. **(C)**, Descriptions of the alignment result. **(D)**, Match information between the query sequence and subject sequences.

BLAST is the most commonly used tool and is included as a separate module in the MacadamiaGGD database. It allows users to perform both BLASTp and BLASTn searches to rapidly align sequences to the database. In the BLAST module, pasting the DNA/protein sequences in the query box or uploading a FASTA file is acceptable. For example, the users can enter “Example 1” sequence in the blank box and select the against database type, e-value, and max target sequence number and then click the “Run” button to obtain the comparison results *via* the “BLASTp” function (**Figure 3A**). In addition, when pulling down the search result interface, a user is presented with all the comparison results (**Figure 3B**), including the description information of the candidate subject sequences alignment parameters (**Figure 3C**) and the matching information between the query sequence and each subject sequence (**Figure 3D**).

### Markers

In the “markers” module, we included 657 SSR markers and 35 SNPs. Macadamia trees have a relatively long juvenile period (commonly four to five years); thus, it would take a great deal of time to select high-yielding cultivars for breeding. Molecular markers that are associated with key yield traits are extremely important for developing rapid cycle breeding programs in macadamia (O'Connor et al., 2020). To verify the polymorphism of SSR markers from previous research (Schmidt et al., 2006; Nock et al., 2014b; Langdon et al., 2019), we randomly selected 8 primer pairs from MacadamiaGGD (**Table S2**) and identified polymorphisms of these SSRs *via* electrophoresis. The results showed that the selected primer pairs were polymorphic.

**Figure 4:**
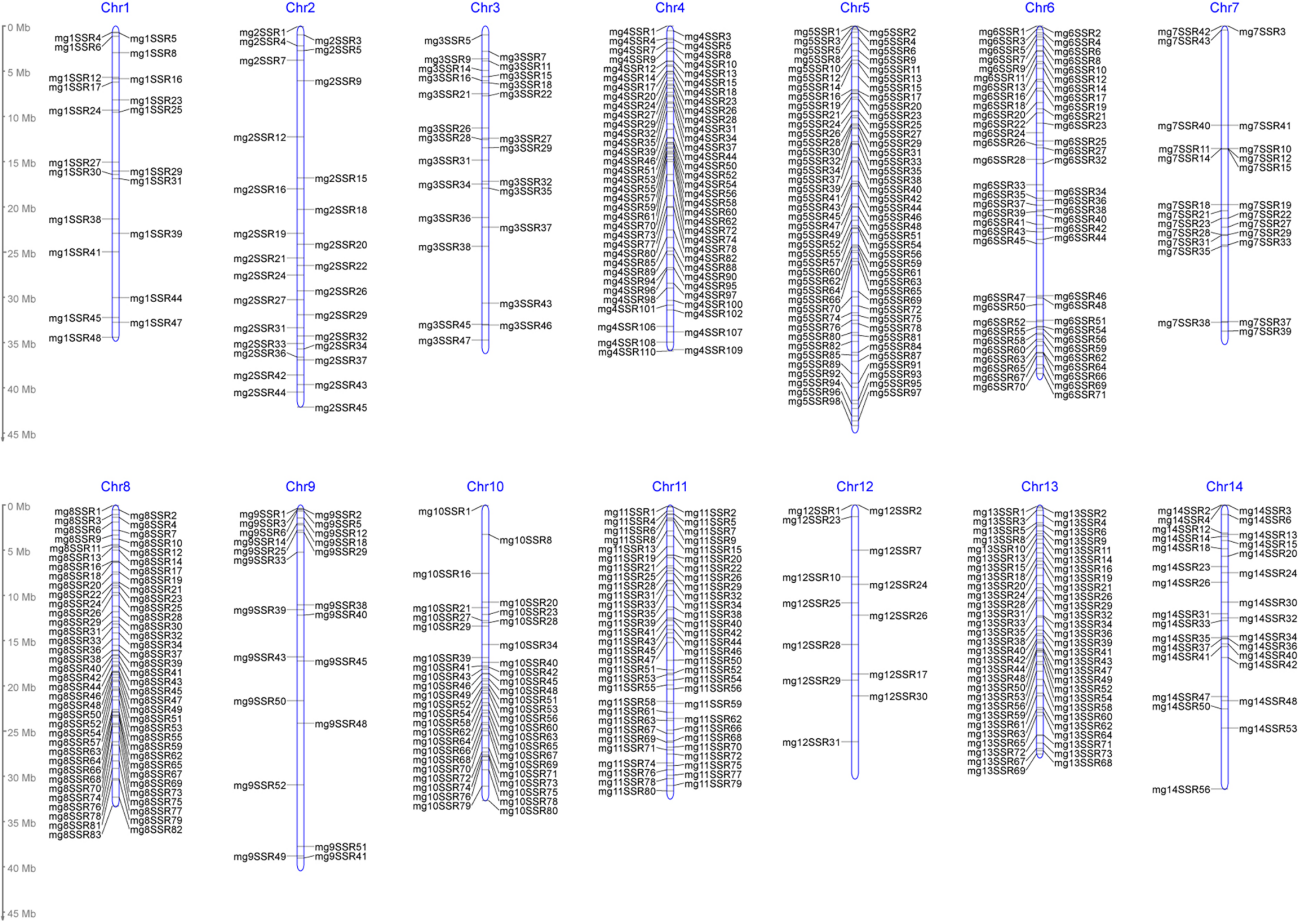
Density distribution map of polymorphic SSR markers on chromosomes in *Macadamia integrifolia*.

In this study, a total of 145593 SSR loci were obtained from *M. integrifolia* HAES 741 genomic sequences (Nock et al., 2020). They were evenly distributed on 14 chromosomes, with an average density of 10400 loci per chromosome (**Table 2**). SSR motifs exist as one of three main types: dinucleotide repeats (DNRs), trinucleotide repeats (TNRs) and tetranucleotide repeats (TTRs). Among these SSRs, DNRs were the most abundant (115139), followed by TNRs (26400) and TTRs (4054), which accounted for 79%, 18% and 3%, respectively (**Table 2**). A total of 927 primer pairs were designed by the selection of the SSR loci with repeat numbers ≥30 from the total SSR loci (**Table S3**). Out of 927 amplified products, 605 primer pairs were polymorphic, with an average of 1.17 SSR markers per Mb on 14 chromosomes. According to the SSR density distribution map, chromosome 5 had the highest number of SSRs (81), but chromosome 12 had only 13 SSRs (**Figure 4**). In addition, a total of 35 SNPs were included in the “markers” module, which were significantly associated with the yield component traits identified by genome-wide association studies (GWASs) (O'Connor et al., 2019a; O'Connor et al., 2020).

**Table 2:** Characterization of the screened SSRs in *Macadamia integrifolia*.

Chromosome	DNR	TNR	TTR	AllSSR loci	Proportion to allSSR loci (%)	All SSRs	SSRs densitydistribution on chromosome(1/Mb)
Chr1	6453	1276	196	7925	5.44	21	0.58
Chr2	10092	2386	352	12830	8.81	28	0.64
Chr3	9217	2048	349	11614	7.98	25	0.66
Chr4	8935	2142	318	11395	7.83	67	1.79
Chr5	11249	2537	400	14187	9.74	81	1.72
Chr6	8208	1823	291	10322	7.1	63	1.54
Chr7	8730	2065	289	11084	7.61	24	0.65
Chr8	8878	1991	288	11157	7.66	73	2.09
Chr9	6890	1566	249	8705	5.98	22	0.52
Chr10	7443	1833	269	9547	6.56	44	1.29
Chr11	7625	1856	271	9752	6.7	59	1.74
Chr12	7315	1666	250	9231	6.34	13	0.41
Chr13	6562	1516	257	8335	5.72	57	1.95
Chr14	7542	1695	275	9512	6.53	29	0.88
Total	115139	26400	4054	145593	100	606	

DNR, dinucleotide repeat; TNR, trinucleotide repeat; TTR, tetranucleotide repeat; Mb, megabase.

### Maps

The map module contains nine genetic linkage maps derived from three macadamia cultivars, HAES 741, HVP A268 and HVP A4. In each map, there were 14 linkage groups (LGs), which correspond to the number of haploid chromosomes in macadamia. When the users open this module, the features of the maps are displayed, including the description and number of maps. The images of the maps appear at the lower left of the module, while the detailed information of the LG location, the marker numbers, the largest and smallest gap, the total length and the average length between markers is displayed at the lower right.

### Tools

The tools module contains several utilities, including “Primer designer”, “Sequence Fetch”, “Enrichment analysis”, “Multiple sequence alignment”, “Genome alignment”, and “Gene homology annotation”, which allow a relatively complete bioinformatics analysis. The user can click the “Primer designer” button, input the nucleic acid sequence or select a scaffold range, adjust the appropriate parameters, and click the “Run” button to obtain a satisfactory pair of primers. Users can screen functional genes of interest (GOIs) based on the data of the *M. integrifolia* transcriptome, click the “Enrichment analysis” button, input the gene ID in the dialog box and select Kyoto Encyclopedia of Genes and Genomes (KEGG) or Gene Ontology (GO) for functional clustering analysis. “Sequence Fetch” can be used to efficiently obtain the sequence of GOI from the *M. integrifolia* genome, which can acquire either a certain or multiple gene sequences at the same time. “Muscle” is a multisequence alignment tool that not only can be used to obtain homology between genes but also can be used to build an intuitive diagram. The “primer designer” tool can be used to design specific primers to clone GOIs for functional research. In addition, by using the “LASTZ” and “GeneWise” tools, users can complete genome alignment and gene homology annotation, respectively.

### References

Currently, the “Reference” module contains the macadamia germplasm and genome-related references, which allows users to query approximately 40 articles information related to the data contained in MacadamiaGGD. The completion and optimization of macadamia genome sequencing results among these publications contribute to the study of macadamia functional genomics and comparative genomics and are convenient for molecular plant breeding efforts.

### A case study involving the use of MacadamiaGGD

**Figure 5:**
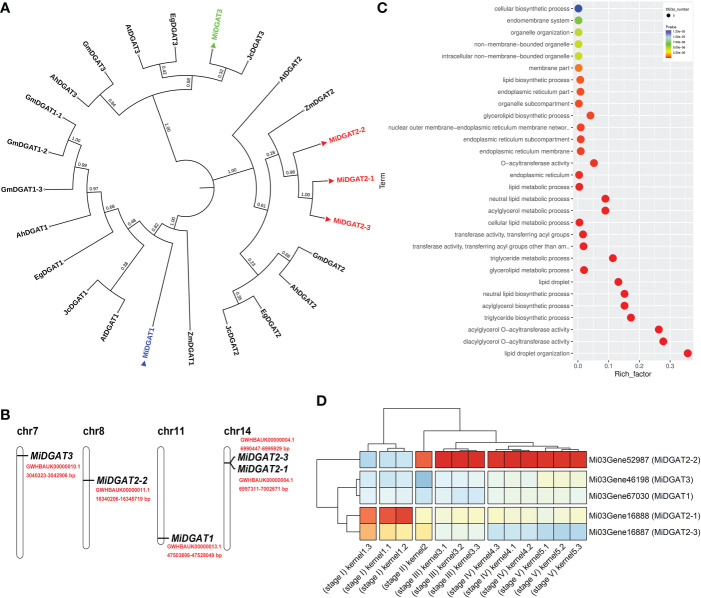
A case study for the application of MacadamiaGGD. **(A)**, Phylogenetic analysis of macadamia MiDGATs and DGATs from other plants. The phylogenetic tree was constructed *via* the neighbor-joining method and 1000 bootstraps by the software MEGA 11 (https://megasoftware.net/). The tree was visualized by iTOL (https://itol.embl.de/). Macadamia MiDGAT proteins and their sequence accessions are MiDGAT1 (Mi03Gene67030), MiDGAT2-1 (Mi03Gene16888), MiDGAT2-2 (Mi03Gene52987), MiDGAT2-3 (Mi03Gene16887) and MiDGAT3 (Mi03Gene46198) from *Macadamia integrifolia*. The proteins and their sequence accessions from other plants are AtDGAT1 (NP_179535), AtDGAT2 (AEE78802) and AtDGAT3 (Q9C5W0.2) from *Arabidopsis thaliana*, GmDGAT1-1 (NP_001237289), GmDGAT1-2 (NP_001237684.2), GmDGAT1-3 (NP_001242457.1), GmDGAT2 (NP_001299586.1) and GmDGAT3 (XP_003542403.1) from *Glycine max*, AhDGAT1 (AGT57761.1), AhDGAT2 (AEO11788.1) and AhDGAT3 (AAX62735.1) from *Arachis hypogaea*, JcDGAT1 (NP_001292926), JcDGAT2 (NP_001292973) and JcDGAT3 (XP_012083005.1) from *Jatropha curcas*, ZmDGAT1 (NP_001349157.1) and ZmDGAT2 (AQL03438.1) from *Zea mays*, and EgDGAT1 (XP_039165824.1), EgDGAT2 (XP_010033619.2) and EgDGAT3 (XP_010024878.2) from *Eucalyptus grandis*. **(B)**, Distribution of *MiDGAT* genes within the macadamia genome. The chromosome number is indicated at the top of each chromosome. The red font indicates the specific physical position of the genes. **(C)**, GO enrichment of *MiDGATs*. **(D)**, Expression pattern of *MiDGAT*s at different developmental stages of macadamia kernels. The transcripts per million (TPM) values of expression levels are graphically represented by the Pheatmap package (R 4.2.0).

MacadamiaGGD integrates BLAST, enrichment analysis, and other tools for functional genomic research of Macadamia. Acyltransferases are the potential molecular targets for genetic engineering to increase the oil content and alter the fatty acid composition in the oil crops (Zhang et al., 2021). Here, we provide a case study on the *diacylglycerol acyltransferases* (*DGAT*s) of *M. integrifolia* by using the “BLAST”, “GO enrichment”, “JBrowse”, and “Gene Expression” function of MacadamiaGGD. By using BLAST in MacadamiaGGD, the Conserved Domains Database (CDD) of the NCBI database, the SMART database (https://smart.embl.de/) and MEGA 11 software (https://megasoftware.net/), we obtain one DGAT1 (MiDGAT1), three DGAT2 (MiDGAT2-1, MiDGAT2-2, MiDGAT2-3), and one DGAT3 (MiDGAT3) (**Figure 5A**). Of the five *MiDGAT* genes, two genes (*MiDGAT2-1*, *MiDGAT2-3*) were mapped to chromosome 14, and their physical positions were very close (**Figure 5B**).

To verify the expression features of *MiDGATs* during triacylglycerol (TAG) biosynthesis, we downloaded the transcriptome expression data of *M. integrifolia* kernel development from MacadamiaGGD. By using gene ontology (GO) annotation information available from MacadamiaGGD, we conducted the GO enrichment analysis of the five *MiDGAT* genes from *M. integrifolia*. The results showed the five *MiDGAT*s were enriched in more than 30 GO terms, which are involved in fatty acid and TAG biosynthesis in plants (**Figure 5C**). Further, we also investigated the expression profile of *MiDGAT*s at five stages of kernel development. *MiDGAT2-1* and *MiDGAT2-3* were highly expressed in stages I and II (**Figure 5D**). *MiDGAT2-2* exhibited low expression levels in stages I and II, whereas it was highly expressed in stages III, IV, and V. Consistent with these results, The expression pattern of *MiDGAT2* was recently found to be mainly correlated with fatty acid biosynthesis at different stages of developing kernels (Gao et al., 2021).

## Discussion

The macadamia database MacadamiaGGD serves as an integrated germplasm and genomic research platform that can facilitate the genomic research and molecular breeding of macadamia. MacadamiaGGD integrates the currently published macadamia datasets of genomes, genetic maps, molecular markers, and morphological data of four macadamia species. MacadamiaGGD consists of 11 functional modules: Home, Germplasm, Genomes, Expression, BLAST, Markers, Maps, Tools, References, Download and Help.

Compared to other existing genome databases, the MacadamiaGGD provides a more comprehensive database and tools to characterize germplasms and genes of macadamia species. For example, “Phylogenetic Analysis”, which is integrated in the Germplasm module of MacadamiaGGD, was not included in the Citrus Genome Database (CGD, https://www.citrusgenomedb.org/), the Rice Genome Hub (RGH, https://rice-genome-hub.southgreen.fr) (Droc et al., 2019), the Kiwifruit Genome Database (KGD; http://kiwifruitgenome.org/) (Yue et al., 2020), and the functional genomics database for cannabis (CannabisGDB, https://gdb.supercann.net) (Cai et al., 2021). Databases of two kinds of molecular markers, SSR and SNP, are included in MacadamiaGGD, but not available in RGH, KGD, CannabisGDB, and the Gossypium Resource and Network Database (GRAND, http://grand.cricaas.com.cn) (Zhang et al., 2022). And MacadamiaGGD provides genetic linkage maps of nine genotypes, whereas genetic linkage maps are not available in KGD, CannabisGDB, and GRAND. Given the comprehensive information, interactive nature, and user-friendly database, MacadamiaGGD makes it easy to retrieve genomic information of macadamia. Thus, MacadamiaGGD not only provides a convenient way for researchers to understand and acquire basic germplasm and genomic information but also can largely help advance the molecular breeding of macadamia in the future.

The macadamia genome was used for the exploration of the SSR motifs, which were found to be evenly distributed across all 14 chromosomes. However, the percentage of the three SSR motifs was different, among which DNRs accounted for 79%, TNRs accounted for 18%, and TTRs accounted for 3%. This pattern is consistent with that in *Myrica rubra* (Jiao et al., 2012), in which DNRs were dominant. In this study, 927 primer pairs were designed for the verification of SSR locus polymorphisms, among which 605 primer pairs were found to be polymorphic. The density of microsatellite distribution was approximately 1.17 SSRs/Mb on 14 chromosomes, which was much higher than that in previous studies (Nock et al., 2014b). The main reason for this discrepancy may be due to the differences in genome quality and the SSR prediction method. In summary, we developed the first database of macadamia germplasm, genome, and genome-based SSR marker information, which will facilitate the molecular breeding of macadamia.

## Conclusion

In conclusion, we developed the first comprehensive macadamia germplasm and genomic database MacadamiaGGD, which could serve as a central portal for macadamia species. MacadamiaGGD integrates data from germplasm, genomes, transcriptomes, genetic linkage maps, and SSR markers from various macadamia species. MacadamiaGGD also provides a group of user-friendly modules that enable users worldwide to efficiently retrieve and analyze genomic data. At present, MacadamiaGGD is in its first version but will be updated in a timely manner when new macadamia germplasm and omics data are available or published. We believe that MacadamiaGGD not only will broaden the understanding of the germplasm, genetics and genomics of macadamia species but also will facilitate the molecular breeding of macadamia.

## Data availability statement

The original contributions presented in the study are included in the article/**Supplementary Material**. Further inquiries can be directed to the corresponding authors.

## Author contributions

Z-FX and JN designed the research. PW, YM, YW, YF, and JH collected and processed genomic and germplasm data. YM and PW developed the SSRs. PW, Z-FX and JN wrote the first draft of this manuscript. All authors contributed to the edit of this manuscript and the construction of MacadamiaGGD. All authors contributed to the article and approved the submitted version.

## Funding

This work was supported by start-up research funds from the Guangxi University.

## Acknowledgments

We would like to thank all the macadamia researchers who have created valuable data resources collected in McadamiaGGD. Thanks to Mr. Zheng Yin and Mr. Zequn Zheng (VGsoft Team, China) for assisting in the website construction.

## Conflict of interest

The authors declare that the research was conducted in the absence of any commercial or financial relationships that could be construed as a potential conflict of interest.

## Publisher’s note

All claims expressed in this article are solely those of the authors and do not necessarily represent those of their affiliated organizations, or those of the publisher, the editors and the reviewers. Any product that may be evaluated in this article, or claim that may be made by its manufacturer, is not guaranteed or endorsed by the publisher.
